# Day Surgery Conversion: Rate and Possible Causes in King Fahad Specialist Hospital, Buraydah, Saudi Arabia

**DOI:** 10.7759/cureus.20790

**Published:** 2021-12-29

**Authors:** Sultan H Alsaigh, Yusra M Aldughaishem, Hareth N Alhujaili, Turky S Alfadda, Majed A Almutairi, Jana I Albulaihi, Renad a Almugbel, Ahmad F Alhumaid

**Affiliations:** 1 General Surgery, King Fahad Specialist Hospital, Buraydah, SAU; 2 College of Medicine, Qassim University, Qassim, SAU

**Keywords:** hernia repair, surgical patients, cholecystectomy, conversion rate, day surgery

## Abstract

Introduction

Day surgery is defined as the admission of a patient and discharge after the surgical procedure within the same day. It is becoming increasingly popular as it provides multiple advantages for the health care system and patients, including better resource utilization in the form of decreasing the cost, increasing the number of patients served, a reduction in the waiting list length, and preservation of hospital beds for complex advanced cases. Internationally, patients' admission rate after a planned day surgery was found at 1.5%. Etiologies for day surgery conversion vary among studies, 75% of which were labeled as potentially preventable. As patients are admitted, the benefits of day surgery decrease.

Aim

This study aimed to measure the unanticipated admission rate of day surgery while evaluating the reasons for admission in King Fahad Specialist Hospital, Buraydah, Qassim, Saudi Arabia.

Materials and methods

This is a retrospective review study in which all medical records of patients admitted and operated as day surgery from January 1, 2015 to February 28, 2021 in King Fahad Specialist Hospital in Buraydah, Qassim region in Saudi Arabia were evaluated. Variables collected included baseline characteristics such as age, gender, body mass index (BMI), chronic diseases, surgical history, operation name, complications, and causes of conversion.

Results

A total of 6,771 day surgery cases were found from January 1, 2015 to February 28, 2021. Of them, 231 cases were converted to inpatient admission, and the prevalence of conversion was 3.4%. The most common cause of conversion was pain (35.1%) followed by postoperative care (16%), need of antibiotics (12.1%), and bleeding (8.2%); most of these cases were associated with laparoscopic cholecystectomy. Furthermore, laparoscopic cholecystectomy (36.4%), hernia repair (12.1%), and pilonidal sinus excision were the most frequent surgical intervention.

Conclusion

The day surgery conversion rate was minimal in this study (3.4%). Pain, postoperative care, and the need for antibiotics were the most common reasons for conversion. Continuous monitoring of day surgery conversion rate and causes will all help the institution to gain the maximum benefits of day surgeries.

## Introduction

Day surgery, also known as ambulatory surgery and same-day surgery, in the United Kingdom (UK), is defined as the admission of a patient and discharge after a surgical procedure within the same day [1,2]. Day surgery is becoming increasingly popular as estimated by the National Health Statistics Reports of the United States of America in 2017, with multiple types of procedures being done as ambulatory [3]. In 2010, for instance, tonsillectomy with or without adenoidectomy, which is one of the commonest procedures in children, has been estimated to be done 339.000 times across the United States (US) [4].

Day surgery provides the health care system with multiple advantages including better resource utilization in the form of decreasing the cost, increasing the number of patients served, a reduction in the waiting list length, and preservation of hospital beds for complex advanced cases [5-8]. Patients also benefit from day surgery; the risk of nosocomial infection will be reduced, mobilization and physical activity are returned to premorbid level earlier, mental disability is reduced as well [1,5,7,9,10]. Meanwhile, their satisfaction, regardless of operation type, was reported to be 95% at the time of discharge and 30 days after [11]. Nevertheless, one of the main factors negatively affected patient satisfaction was postoperative pain management [11]. However, 97% of patients will choose it again according to Beverly Philip’s study [12].

Ambulatory surgery is appropriate for most patients. However, social, medical and surgical factors should not be overlooked. Socially, patient understanding and approval of the day surgery, with the presence of a carer if indicated for a suitable time post-operatively, geographical distance with traveling time less than one hour to the hospital. Medically, according to patient functional status and stability of chronic diseases if present. Surgically, the patient should not be going through a surgical procedure with a possible complication that requires immediate medical attention. Generally, it is recommended to collaborate in a multidisciplinary approach with local surgeons and anesthetists keeping patient safety into consideration for day cases assessment [1,2,7].

Internationally, patients' admission rate after a planned day surgery was found to be 1.5% in a study done in 2002, with ENT being the highest percentage of admission 2.5% [13]. Yet in 2018, 2.89% were admitted due to various causes irrespective of the type of surgery [14]. Etiologies for admission vary between studies, 75% of which were labeled as potentially preventable reasons for admission [13]. However, the most recent study found the following causes as the highest ones: patients requiring more extensive surgery, severe pain, or bleeding as surgical causes. While organizational/social causes were as follows: late start operation, lack of home support, patient or surgeon’s request were also recognized as causes [14]. Nationwide, out of 487 patients who underwent laparoscopic cholecystectomy, 22 were admitted, primarily for abdominal pain, conversion to open surgery, or persistent pain [15]. Another study done in Bisha reported that 25 out of 224 patients were admitted with planned one-day laparoscopic cholecystectomy [16].

With patients being admitted, the benefits of day surgery to the health care system and patients decrease. Studies have shown variation in admission rates for different surgical specialties with various etiologies. In this study, we measured the unanticipated admission rate of day surgery while evaluating the reasons for admission In King Fahad Specialist Hospital, Buraydah, Qassim, Saudi Arabia.

The objectives of this study are to assess the rate of conversion of day surgery cases to inpatient wards and to specify the different reasons and factors that lead to conversion.

## Materials and methods

Our study design is a retrospective review in which all medical records of patients admitted and operated as day surgery cases were evaluated from January 1, 2015 until February 28, 2021 in King Fahad Specialist Hospital, which is the largest hospital in Buraidah, the capital of Al-Qassim region in Saudi Arabia with a population of approximately 600,000 people. The study was approved by the Regional Research Ethics Committee - Qassim Province (1441-1864435). We included all patients admitted to the day surgery unit and operated as day surgery regardless of age, sex, type of surgery. Any patient who had planned admission was excluded. Regarding sample size, we included all patients who met the criteria in the chosen period, which were 6771 patients. For any patient who met the criteria, we reviewed his medical records to find if he had an unanticipated conversion to inpatient wards.

Statistical analysis

Statistical Package for the Social Sciences (SPSS) version 26 (IBM Corp., Armonk, New York) was used to analyze data. Descriptive statistics were presented using numbers and percentages.

## Results

This study involved 231 patients. As seen in Table 1, the most common age group was 35 years old or less (55.8%), with nearly 60% being males. Obese patients constitute 35.4%, while overweight patients constitute 32.6%. The most commonly detected chronic diseases were diabetes + hypertension (4.3%), followed by diabetes alone (3.5%) and hypertension (2.2%). Furthermore, the most frequently reported surgical history was hernia repair (3%) and appendectomy (3%). Likewise, the most commonly performed operation was laparoscopic cholecystectomy (36.4%), followed by hernia repair (12.1%) and pilonidal sinus excision (10%), while septoplasty was the least (1.7%). Additionally, the most common complication of operation was bleeding (1.3%). The proportion of patients who reported complications of anesthesia was 0.9% (n=2).

**Table 1:** Baseline and clinical characteristics of the patients (n=231) BMI: body mass index; DM: diabetes mellitus; IDA: iron deficiency anemia; TB: tuberculosis; GI: gastrointestinal.

Study Variables	Number (%)
Age group	
≤35 years	129 (55.8%)
>35 years	102 (44.2%)
Gender	
Male	133 (57.6%)
Female	98 (42.4%)
BMI ^(n=175)^	
Underweight (<18.5 kg/m2)	10 (05.7%)
Normal (18.5 – 24.9 kg/m2)	46 (26.3%)
Overweight (25 – 29.9 kg/m2)	57 (32.6%)
Obese (≥30 kg/m2)	62 (35.4%)
Associated chronic diseases	
None	189 (81.8%)
DM + Hypertension	10 (04.3%)
Diabetes Mellitus	08 (03.5%)
Hypertension	05 (02.2%)
Asthma	04 (01.7%)
Hypothyroidism	03 (01.3%)
Mental disorder	03 (01.3%)
Others (IDA, down syndrome, old TB, etc.)	09 (03.9%)
Surgical history	
None	197 (85.3%)
Hernia repair	07 (03.0%)
Appendectomy	07 (03.0%)
Sleeve gastrectomy	05 (02.2%)
Tonsillectomy	02 (0.90%)
Others (Fracture surgery, laser hemorrhoids, amputation)	13 (05.6%)
Operation name	
Laparoscopic cholecystectomy	84 (36.4%)
Hernia repair	28 (12.1%)
Pilonidal sinus excision	23 (10.0%)
Ureteroscopy	22 (09.5%)
Teeth extraction	13 (05.6%)
Tonsillectomy	08 (03.5%)
Hemorrhoidectomy	05 (02.2%)
Septoplasty	04 (01.7%)
Others (Cystoscopy, Colostomy, Stent removal, etc.)	44 (19.0%)
Operation complication	
None	212 (91.8%)
Bleeding	03 (01.3%)
Difficult surgery	02 (0.90%)
Acute cholecystitis	02 (0.90%)
Intraoperative GI leak/spill	02 (0.90%)
Others (hematoma, urinary retention, jaundice, etc.,)	10 (04.3%)
Anesthesia complication	
Yes	02 (0.90%)
No	229 (99.1%)

**Figure 1:**
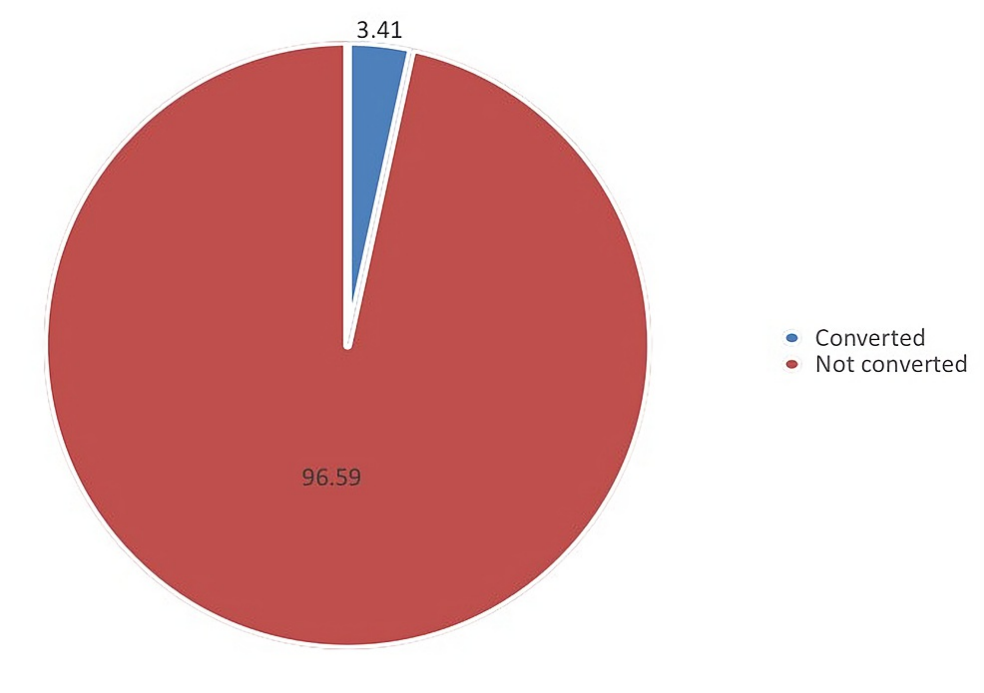
Prevalence of day surgery conversion in six years (2015–2021)

Figure 1 presents the prevalence of day surgery conversion rate in six years. It can be observed that the prevalence of day surgery conversion rate was 3.4%, and the rest were not converted (96.6%).

**Figure 2:**
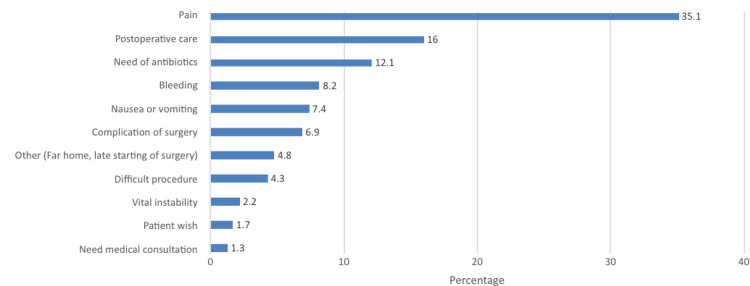
Causes of conversion

In Figure 2, the most common cause of conversion was pain (35.1%), followed by postoperative care (16%), need of antibiotics (12.1%), bleeding (8.2%), and nausea or vomiting (7.4%). while the need for medical consultation was the least (1.3%).

In Table 2, it was observed that pain (35.8%), nausea and vomiting (64.7%), a complication of surgery (43.8%), vital instability (60%), complex procedure (60%), need of antibiotics (e.g. intraoperative gastrointestinal leak/spill) (32.1%) and patients request (50%) were the most commonly complained after laparoscopic cholecystectomy. While bleeding (42.1%) and postoperative care (including vital signs monitoring, post-operative drains, peri-operative events necessitating further assessment) were the causes of conversion due to other types of operation.

**Table 2:** Causes of conversion in accordance to the most commonly performed operation (n=231) N: number; Lap Chole: laparoscopic cholecystectomy.

Causes of Conversion	Lap Chole N (%)	Hernia Repair N (%)	Pilonidal Excision N (%)	Ureteroscopy N (%)	Teeth Extraction N (%)	Tonsillectomy N (%)	Hemorrhoidectomy N (%)	Septoplasty N (%)	Others N (%)
Pain	29 (35.8%)	19 (23.5%)	05 (06.2%)	08 (09.9%)	05 (06.2%)	02 (02.5%)	03 (03.7%)	01 (01.2%)	09 (11.1%)
Postoperative care	08 (21.6%)	03 (08.1%)	04 (10.8%)	04 (10.8%)	03 (08.1%)	01 (02.7%)	02 (05.4%)	01 (02.7%)	11 (29.7%)
Need of antibiotics	09 (32.1%)	02 (07.1%)	02 (07.1%)	6 (21.4%)	01 (03.6%)	02 (07.1%)	0	01 (03.6%)	05 (17.9%)
Bleeding	04 (21.1%)	0	04 (21.1%)	01 (05.3%)	0	01 (05.3%)	0	01 (05.3%)	08 (42.1%)
Nausea or vomiting	11 (64.7%)	0	02 (11.8%)	0	0	0	0	0	04 (23.5%)
Complication of surgery	07 (43.8%)	02 (12.5%)	01 (06.2%)	01 (06.2%)	01 (06.2%)	01 (06.2%)	0	0	03 (18.8%)
Vital instability	03 (60.0%)	01 (20.0%)	0	0	01 (20.0%)	0	0	0	0
Complex procedure	06 (60.0%)	0	01 (10.0%)	01 (10.0%)	01 (10.0%)	0	0	0	01 (10.0%)
Patient wish	02 (50.0%)	0	0	0	0	01 (25.0%)	0	0	01 (25.0%)
Need medical consultation	01 (33.3%)	0	01 (33.3%)	0	0	0	0	0	01 (33.3%)
Others	04 (36.4%)	01 (09.1%)	03 (27.3%)	01 (09.1%)	01 (09.1%)	0	0	0	01 (09.1%)

## Discussion

This study was carried out to examine the rate of day surgery conversion and identify its possible causes. The rate of conversion in this study was 3.4%. While it was reported to be 1.5% in 2002 in Singapore, and 2.89 in Belgium in 2019 [13,14]. Interestingly, other studies concerned only with cholecystectomy have shown a high conversion rate, 11% out of 224 patients, and 4% out of 1140 patients [16,17]. Our study included 36.4% of patients who underwent laparoscopic cholecystectomy which might explain the high conversion rate we found.

Furthermore, we have learned that the primary cause of conversion was pain (35.1%), followed by postoperative care (16%), need of antibiotics (12.1%), bleeding (8.2%), and nausea or vomiting (7.4%). In a study conducted by Tham and Koh, they reported that most of the unplanned admission were surgically related (62.8%), followed by anesthesia (12.2%), social (9.5%), and medical reasons (8.1%) [13]. In Pakistan, reports indicated that patients' observation indicated for various reasons and patient requests were the most common causes of conversion, while in Korea, surgeon requests and patients' wishes were the significant causes of conversion [18,19]. In our study, only 1.7% indicated patient wish as the cause of conversion and is the second least identified cause which did not concur with previous results.

Moreover, we noted that laparoscopic cholecystectomy was the most common surgical intervention (36.4%), followed by hernia repair (12.1%) and pilonidal sinus excision (10%). In terms of complications, bleeding and complex surgery were the major surgical complications. Mihailescu et al. documented that 22 out of 598 ambulatory surgery patients demonstrated complications, and out of 22 patients, 11 were converted to conventional hospitalization due to medical, surgical and organizational reasons [20]. On the contrary, Al-Omani and colleagues reported that there was no major complication being reported among patients who underwent laparoscopic cholecystectomy during the five-year experience [17].

Incidentally, we came to know that pain, nausea or vomiting, complication of surgery, vital instability, complex procedure, the need for antibiotics (e.g. intraoperative gastrointestinal leak/spill), and patient wish were the most common complaints after laparoscopic cholecystectomy while bleeding and postoperative care could likely be associated with complaints with other types of surgery. In Singapore, the majority of unplanned admissions were due to common problems like postoperative pain, admission for surgical observation, and social reasons. Furthermore, they remarked that 25% of the non-preventable causes were mainly due to other medical problems unrelated to the surgical intervention [13]. In contrast, in our study, most of these causes of conversion were unpreventable.

In regard to the previous history of surgical intervention, our investigations showed, 14.7% had a previous history of operation, with hernia repair and appendectomy the most commonly performed surgical intervention. Likewise, 18.2% identified having associated chronic diseases, diabetes mellitus and hypertension were dominant. In addition, we noted that two patients had anesthesia complications, which were also contributed to an unintended admission rate.

Although our study highlighted the causes of conversion in the largest center in the region, the retrospective nature of the study with the possibility of missing data due to documination related issues might affect the results accuracy, and a larger multi-central study is required with the aim of identifying all the preventable causes and proposing methods to decrease it.

## Conclusions

The day surgery conversion rate was minimal in this study (3.4%). Pain, postoperative care, and the need for antibiotics were the most common reasons for conversion. The causes of unplanned admission can be lowered from a healthcare institution by careful patient selection (absence of comorbidities, living nearby), prioritizing day surgery cases in the operating room, and better pain management all of which could contribute to a lower conversion rate. Continuous monitoring of day surgery conversion rate and causes will all help the institution to gain the maximum benefits of day surgeries.
